# Succession of endophytic fungi and arbuscular mycorrhizal fungi associated with the growth of plant and their correlation with secondary metabolites in the roots of plants

**DOI:** 10.1186/s12870-021-02942-6

**Published:** 2021-04-05

**Authors:** Hanli Dang, Tao Zhang, Zhongke Wang, Guifang Li, Wenqin Zhao, Xinhua Lv, Li Zhuang

**Affiliations:** College of life Sciences, Shihezi University, Shihezi City, 832003 Xinjiang China

**Keywords:** Arbuscular mycorrhizal fungi, Endophytic fungi, High-throughput sequencing, Medicinal licorice, Plant growth, Secondary metabolites

## Abstract

**Background:**

To decipher the root and microbial interaction, secondary metabolite accumulation in roots and the microbial community’s succession model during the plant’s growth period demands an in-depth investigation. However, till now, no comprehensive study is available on the succession of endophytic fungi and arbuscular mycorrhizal fungi (AMF) with roots of medicinal licorice plants and the effects of endophytic fungi and AMF on the secondary metabolite accumulation in licorice plant’s root.

**Results:**

In the current study, interaction between root and microbes in 1–3 years old medicinal licorice plant’s root and rhizospheric soil was investigated. Secondary metabolites content in licorice root was determined using high-performance liquid chromatography (HPLC). The composition and diversity of endophytic and AMF in the root and soil were deciphered using high-throughput sequencing technology. During the plant’s growth period, as compared to AMF, time and species significantly affected the diversity and richness of endophytic fungi, such as Ascomycota, Basidiomycota, *Fusarium*, *Cladosporium*, *Sarocladium*. The growth period also influenced the AMF diversity, evident by the significant increase in the relative abundance of *Glomus* and the significant decrease in the relative abundance of *Diversispora*. It indicated a different succession pattern between the endophytic fungal and AMF communities. Meanwhile, distance-based redundancy analysis and Mantel tests revealed root’s water content and secondary metabolites (glycyrrhizic acid, liquiritin, and total flavonoids), which conferred endophytic fungi and AMF diversity. Additionally, plant growth significantly altered soil’s physicochemical properties, which influenced the distribution of endophytic fungal and AMF communities.

**Conclusions:**

This study indicated a different succession pattern between the endophytic fungal and AMF communities. During the plant’s growth period, the contents of three secondary metabolites in roots increased per year, which contributed to the overall differences in composition and distribution of endophytic fungal and AMF communities. The endophytic fungal communities were more sensitive to secondary metabolites than AMF communities. The current study provides novel insights into the interaction between rhizospheric microbes and root exudates.

**Supplementary Information:**

The online version contains supplementary material available at 10.1186/s12870-021-02942-6.

## Background

Medicinal licorice belongs to perennial Fabaceae herbs, which grow in arid and semi-arid regions [1]. Three indigenous licorice plants mentioned in Chinese Pharmacopeia are *Glycyrrhiza uralensis*, *Glycyrrhiza inflata,* and *Glycyrrhiza glabra* [2]. Secondary metabolites in *Glycyrrhiza* root, such as polysaccharides, triterpene saponins, and flavonoids in *Glycyrrhiza* root, have profound medicinal use [3, 4]. Glycyrrhizic acid is the most abundant component of triterpenoid saponins [5] and a valuable, pharmacologically active compound, which possesses anti-inflammatory [6], anti-viral, and immunoregulatory properties [5, 7, 8]. Liquiritin is a crucial component of flavonoids with anti-oxidant, anti-bacterial, and anti-inflammatory properties [9, 10].

Licorice has been increasingly used as a health additive, flavoring agent in medicines, foods, and cosmetics, and it is highly popular across the globe [11]. Natural resources, including wild licorice, are rapidly exhausting due to human activities. Thus, cultivated licorice serves as the primary source for licorice root’s active substances [12, 13]. Plants and microbial interaction in the rhizosphere and its surrounding environment plays a crucial role in many ecological processes, such as nutrient cycling and carbon sequestration processes [14]. Endophytes, specifically endophytic fungi, asymptomatically colonizes different tissues of healthy plants, such as stem, leaf, and roots [15]. It plays a crucial role in the host plant’s development and physiology. Apart from providing nutrients and water, endophytes also influence the host’s physiological processes; for instance, endophytes increase stress tolerance and root growth [16]. Endophytic fungi had developed mutualistic interaction with host plants as part of the evolutionary process [17]. Besides, it significantly affects secondary metabolite production and accumulation. Thus, endophytes remarkably impact the medicinal plants’ quality and quantity and the content of medicinal components [17, 18]. To our knowledge, till now, no comprehensive study is available on the association of endophytic fungi with roots of medicinal licorice plants and the effects of endophytic fungi on the secondary metabolite accumulation in licorice plant’s root.

Arbuscular mycorrhizal fungi (AMF), a predominant root symbiote, are classified as a single system called Glomeromycota clade [19]. AMF shows a symbiotic relationship with more than 80% of the terrestrial plants, and it is received carbon from the host plants [20, 21], while increases the nutrient (nitrogen and phosphorus) absorption by the host plant (nitrogen and phosphorus), thus, affects the secondary metabolism of host plants, and enhances the host plant’s tolerance to pathogens, which is directly or indirectly related to plant’s defense system [22, 23]. Symbiosis affects plant-to-plant interactions and the structure of plant communities on a larger scale. Thus, it affects agricultural production as well as the protection and restoration of agricultural ecosystems.

Microbial communities’ temporal dynamics, including the root-related microbiome, are affected by numerous factors, such as pH, nitrogen, and phosphorus content of the soil [24]. In this context, to understand the effects of altered soil characteristics on secondary metabolite, we were investigated in licorice plant during its growth period. Therefore, unraveling the involvement of soil variables in the regulatory mechanism of secondary metabolite accumulation in root and related microbial diversity in licorice plants is crucial to optimize plant-soil interaction in licorice plants cultivation.

High-throughput sequencing (HTS) technology facilitates fast and accurate identification of microbes, including bacteria, fungi, and AMF in various ecosystems as against the traditional culture-dependent techniques [25, 26]. Thus, it broadens the application and understanding of microbial diversity in the ecosystem. In this study, licorice’s rhizospheric soil and root samples were collected from 1 to 3 years old licorice plants, and soil’s physicochemical factors were evaluated to elucidate the effects of secondary metabolites on medicinal plant’s microbial diversity. HTS was employed to investigate the diversity and structure of endophytic fungi and the AMF community in licorice root. We hypothesized that the levels of secondary metabolites in the root increased with time, and it regulated the composition of endophytic fungal and AMF communities in the licorice root. This study aimed to 1) elucidate the changes and correlation between secondary metabolite’s concentration and soil characteristics of three licorice species, 2) explore the succession, composition, and diversity of endophytic fungal and AMF community associated with licorice root, and 3) reveal the interaction between secondary metabolites and microbial community, during 1–3 years of the growth period.

## Results

### Analysis of secondary metabolites in the licorice roots

**Fig. 1:**
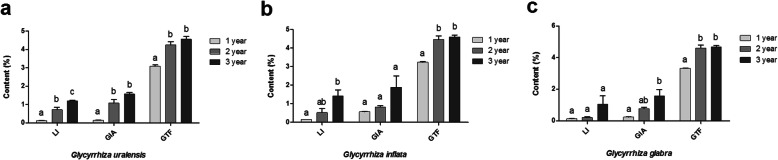
Secondary metabolites content in the root of three licorices species changed with the growth period. Description: Bar charts (mean with standard error) with different lower-case letters represented a significant difference (*P* < 0.05) was assessed by one-way analysis of variance followed by Bonferroni’s statistic test for multiple comparisons. The same letter indicates no significant difference (*P* > 0.05). Abbreviations: GIA, GTF and LI mean glycyrrhizic acid, liquiritin and total flavonoid, respectively

As per the variance analysis, secretion of secondary metabolites differed significantly with the plant’s growth period. As depicted in Fig. 1, liquiritin (LI) content in *G. uralensis* roots increased significantly with the growth period (*P* < 0.05), besides, glycyrrhizic acid (GIA) and total flavonoids (GTF) content increased significantly during 2nd and 3rd year as compared to 1st year (*P* < 0.05) of the growth period. *G. inflata* root showed a significantly higher LI content in the 3rd year than the 1st year (*P* < 0.05) of the growth period, besides, GTF content increased significantly during 2nd and 3rd year as compared to 1st year (*P* < 0.05) of the growth period. Although *G. inflata* root’s GIA content increased with time, no significant differences (*P* > 0.05) were observed. During the three years of the growth period, the LI content increased in the *G. glabra* root, but the difference in LI’s content during the three-year growth period was not significant (*P* > 0.05). GIA content was significantly higher in 3rd year than 1st year (*P* < 0.05), and GTF content was significantly higher in the 2nd and 3rd year (*P* < 0.05) than the 1st year of the growth period. In conclusion, the content of secondary metabolites in the three licorice species’ roots increased significantly during the growth period but to a different extent.

Additionally, as per the variance analysis, physicochemical properties changed significantly with the growth period of licorice plants (Table 1) (*P* < 0.05). Total potassium (STK) content in the 3rd year decreased significantly as compared to 2nd year (*P* < 0.05). Also, total salt (TS) and nitrate nitrogen (SNN) content decreased significantly in 2nd and 3rd year than the 1st year (*P* < 0.05), conversely, ammonium nitrogen (SAN) content increased significantly in the 2nd and 3rd year than the 1st year (*P* < 0.05) of the growth period. Pearson correlation analysis showed that secondary metabolites (GIA, GTF, and LI) were significantly and positively correlated to SAN (r > 0; *P* < 0.05), but significantly and negatively correlated to TS (r < 0; *P* < 0.05) (Table 2), which were consistent with the analysis of stepwise multiple linear regression model between secondary metabolites and soil factors (Fig. S1).

**Table 1:** Effect of growth years on the soil physical and chemical properties

	Variables	1 year	2 year	3 year
Soil Physicochemical Properties	SOM (g/kg)	4.477 ± 0.496	5.186 ± 0.573	4.835 ± 0.718
	STN (g/kg)	0.382 ± 0.070	0.442 ± 0.047	0.403 ± 0.054
	STP (g/kg)	0.435 ± 0.029	0.498 ± 0.041	0.434 ± 0.024
	STK (g/kg)	**22.220 ± 0.466ab**	**23.519 ± 0.397a**	**21.253 ± 0.359b**
	TS (g/kg)	**8.339 ± 0.984a**	**1.372 ± 0.229b**	**3.753 ± 0.542b**
	SNN (mg/kg)	**15.492 ± 1.027a**	**4.389 ± 0.466b**	**9.036 ± 2.098b**
	SAN (mg/kg)	**3.711 ± 0.225a**	**6.217 ± 0.697b**	**5.517 ± 0.426b**
	SAP (mg/kg)	4.437 ± 1.104	2.899 ± 0.337	5.272 ± 0.908
	SAK (mg/kg)	102.452 ± 12.736	128.569 ± 8.422	96.456 ± 10.658
	SWC (%)	3.574 ± 0.380	3.438 ± 0.349	2.795 ± 0.236
	PH	8.462 ± 0.072	8.429 ± 0.087	8.389 ± 0.056

Description: Value is Mean ± standard error, different lower-case letters represented a significant difference (*P* < 0.05) was assessed by one-way analysis of variance followed by Bonferroni’s statistic test for multiple comparisons, the same letter indicates no significant difference (*P* > 0.05)

**Table 2:** Pearson correlation analysis of the content of secondary metabolites with soil physicochemical properties

	GIA	GTF	LI
SOM	0.317	0.172	0.274
STN	0.243	0.071	0.202
STP	0.248	0.101	0.205
STK	−0.156	0.033	−0.251
SNN	−0.270	−0.625^**^	− 0.171
**SAN**	0.472^*^	0.562^**^	0.438^*^
SAP	0.347	−0.023	0.440^*^
SAK	−0.160	0.101	−0.246
**TS**	−0.525**	−0.716**	− 0.383*
PH	−0.075	− 0.218	−0.048
SWC	−0.311	−0.092	− 0.381

Description: the values are the Pearson correlation coefficients. The correlation coefficient r of Pearson is between −1 and 1, r < 0 is negative correlation, r > 0 is positive correlation. ** means *P* < 0.01; * means *P* < 0.05

### The distribution difference between endophytic fungal and AMF communities

**Fig. 2:**
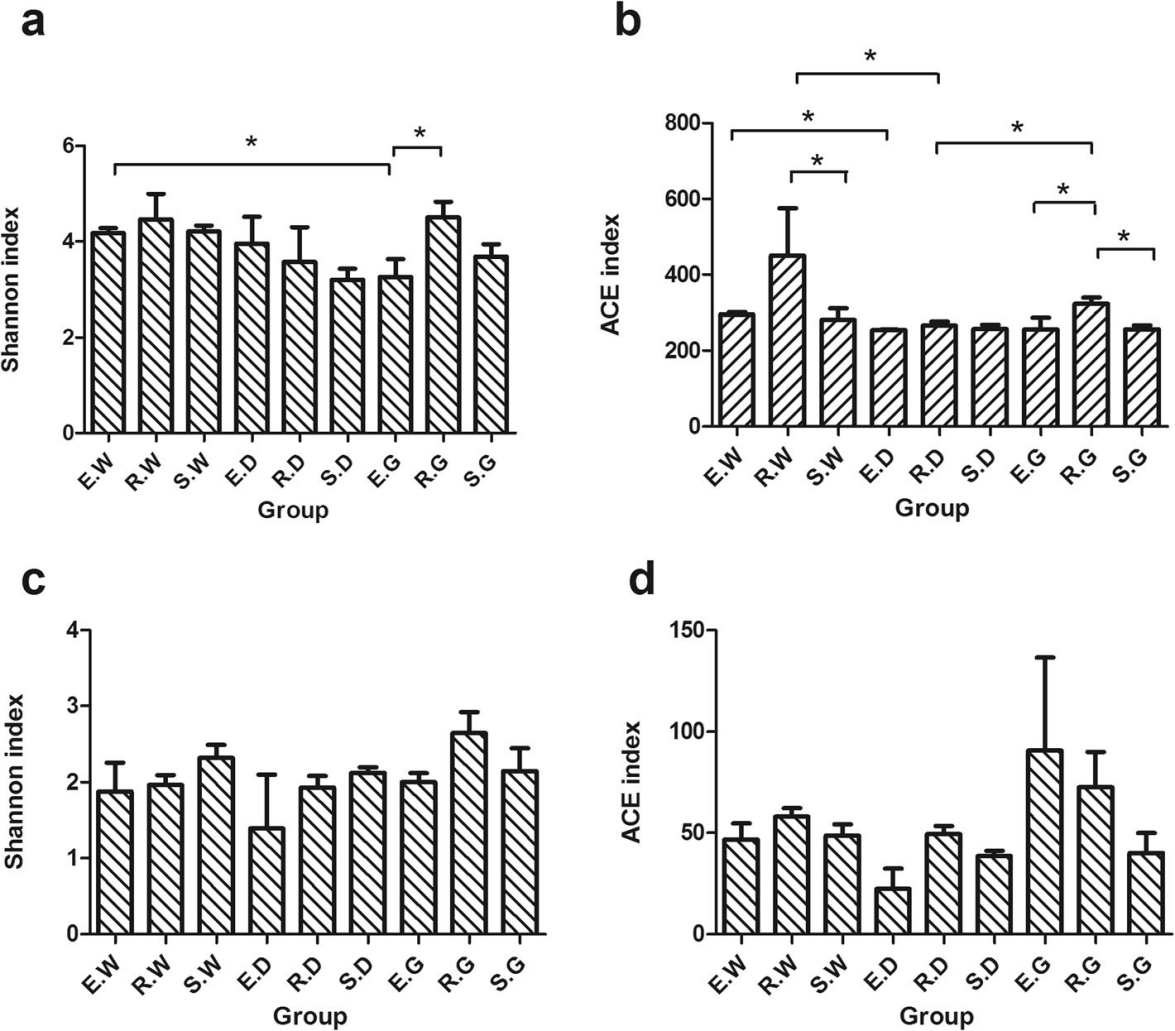
An analysis of Alpha diversity indices based on Wilcoxon rank-sum test. Description: The * represented a significant difference (*p* < 0.05) assessed by Wilcoxon rank-sum test for analysis. Ordinate is Alpha diversity index (Shannon index and ACE index), where (**a**) (**b**) represents the endophytic fungal community and (**c**) (**d**) represents the AMF community. Abscissa is the group name (E, R and S: years 1, 2, and 3, respectively; W, G and D: *Glycyrrhiza uralensis*, *Glycyrrhiza glabra*, and *Glycyrrhiza inflata*, respectively)

As per the Wilcoxon rank-sum test, the alpha diversity indices of endophytic fungal communities between different groups differed significantly (Fig. 2). Specifically, the Shannon index of R.G. samples was significantly higher than E. G samples, and the Shannon index of E. W samples was significantly higher than E. G samples (*P* < 0.05) (Fig. 2a). The ACE index of the R.G. samples was significantly higher than E.G. and S.G. samples and the ACE index of E. W samples was significantly higher than E. D samples. Besides, the ACE index of R. G and R. W samples was significantly higher than R. D samples, and the ACE index of R. W samples was significantly higher than S. W samples (*P* < 0.05) (Fig. 2b). The alpha diversity indices of the AMF community in three licorice species were identical (Fig. 2c, d). It showed that the growth years and species significantly affected the diversity and richness of the endophytic fungal community compared to that of the AMF community.

**Fig. 3:**
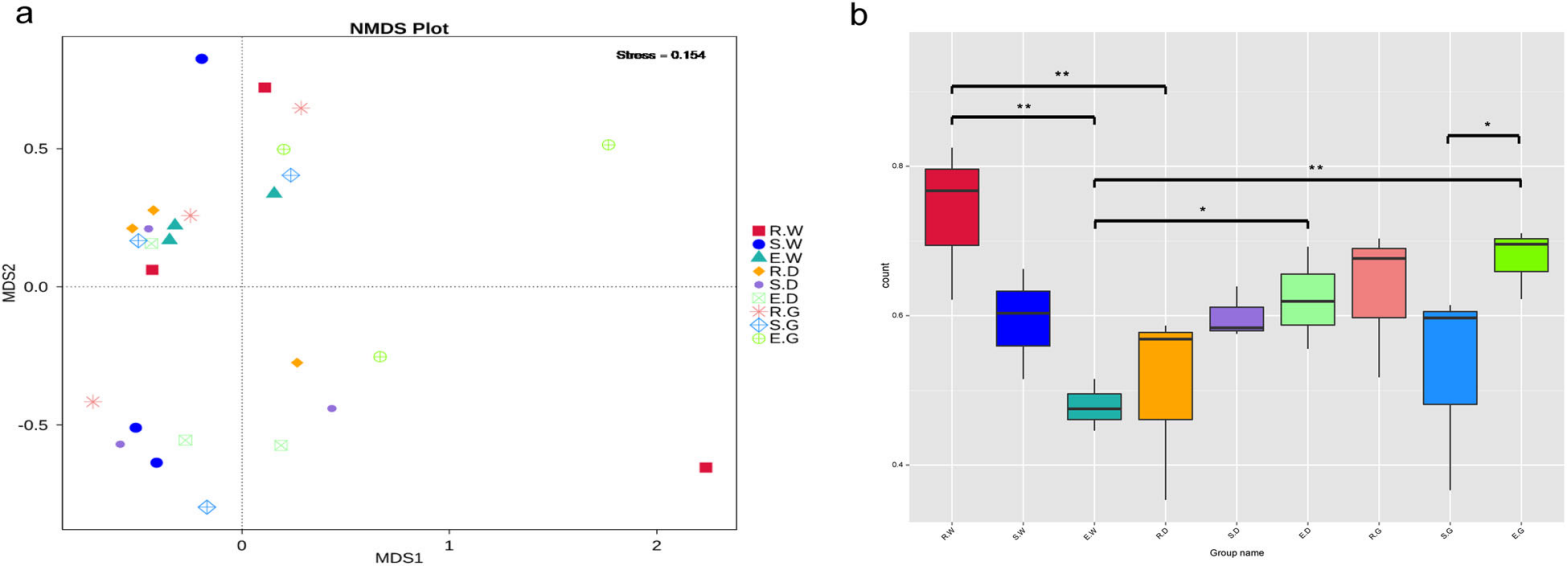
Beta diversity analysis of endophytic fungal community based on weighted UniFrac distance. Description: (**a**) Non-Metric Multi-Dimensional Scaling (NMDS) analysis, which each point in the diagram represents a sample, and samples from the same group are represented in the same color. The lower Stress (< 0.2) indicates that NMDS can accurately reflect the degree of difference between samples. (**b**) The significance test of the differences of Beta diversity, which the * represented a significant difference (*p* < 0.05) assessed by Wilcoxon rank-sum test for analysis. Ordinate is the Beta diversity; abscissa is the group name that has identical meanings as described in Fig. 2

Non-metric multidimensional scaling (NMDS) analysis showed that the endophytic fungi in each sample were isolated from each other. It indicated that the growth period and species significantly influenced the composition of the endophytic fungal community (Fig. 3a). The stress of NMDS analysis was 0.154, which indicated the accuracy of the statistical method (Fig. 3a). NMDS analysis validated the outcome of beta diversity indices. As per Weighted-unifrac based Wilcox rank-sum test composition of endophytic fungal communities differed significantly between R. W and E. W samples (*P* < 0.01), E. G and S. G samples (*P* < 0.05), R. W and R. D samples (*P* < 0.01), E. D and E. W samples (*P* < 0.05), and E. G and E. W samples (*P* < 0.01) (Fig. 3b).

**Fig. 4:**
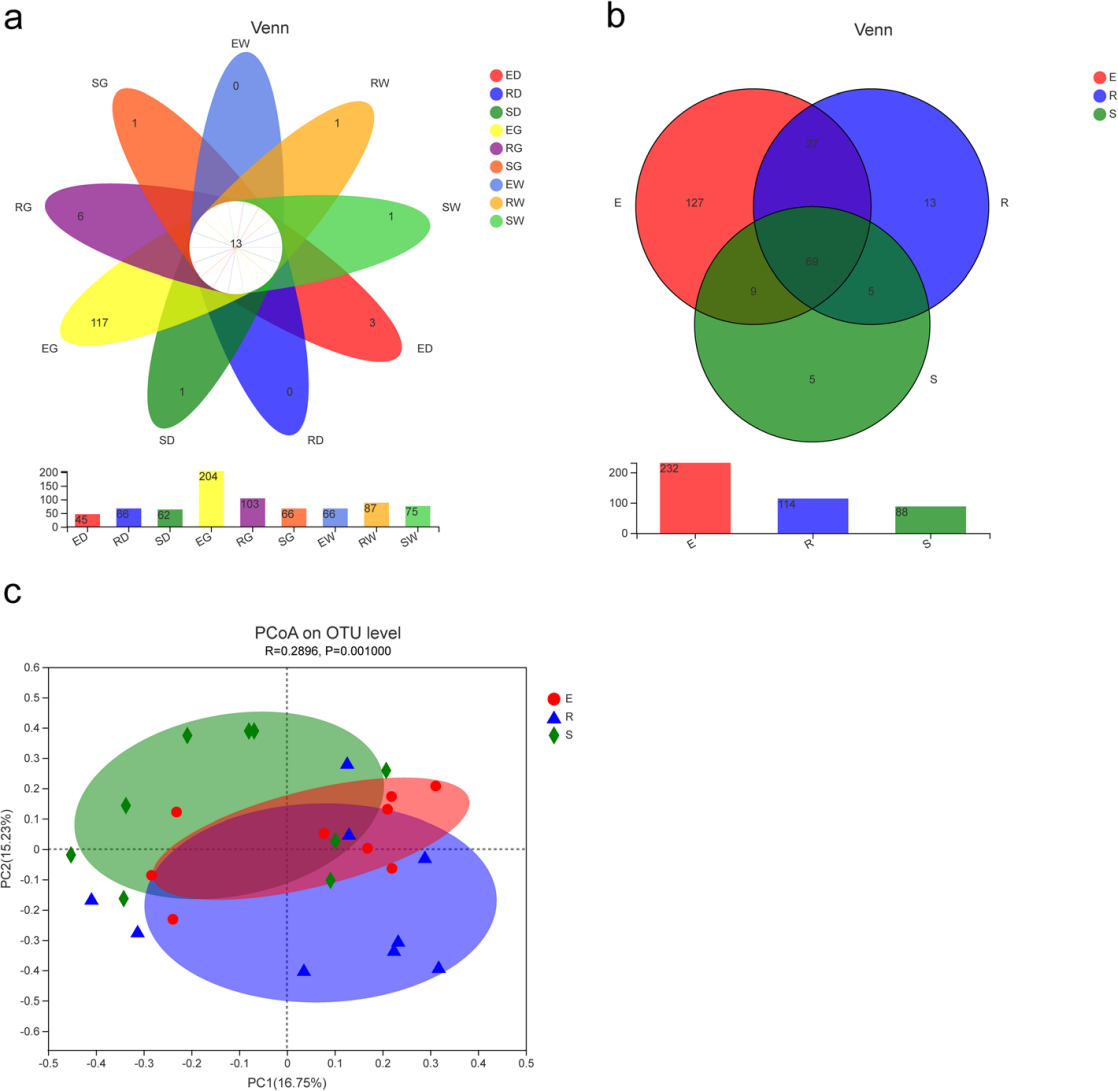
Distribution difference of AMF community. Description: The petal diagram (**a**) and Venn diagram (**b**) based on operational taxonomic units (OTU), which represent common or unique OTUs to a given group. Group name has identical meanings as described in Fig. 2. The Principal Co-ordinates Analysis (PCoA) plot based on weighted unifrac distances for year 1, 2, 3 group (**c**), which each point in the diagram represents a sample, and samples from the same group are represented in the same color

As depicted in the petal diagram, 13 common OTUs were present in each sample’s AMF community (Fig. 4a). The number of AMF OTUs in rhizospheric soil reduced significantly with the growth period, and the highest number of unique OTUs occurred during the 1st year (127) and lowest in the 3rd year (5) (Fig. 4b) of the growth period. Moreover, as per the principal coordinate analysis (PCoA) based on the Bray-Curtis distance algorithm, licorice species did not affect the composition of AMF species significantly; however, the growth period significantly altered the AMF species composition (R = 0.2896, *P* = 0.001) (Fig. 4c).

### Differences in the composition of endophytic fungal and AMF communities

**Fig. 5:**
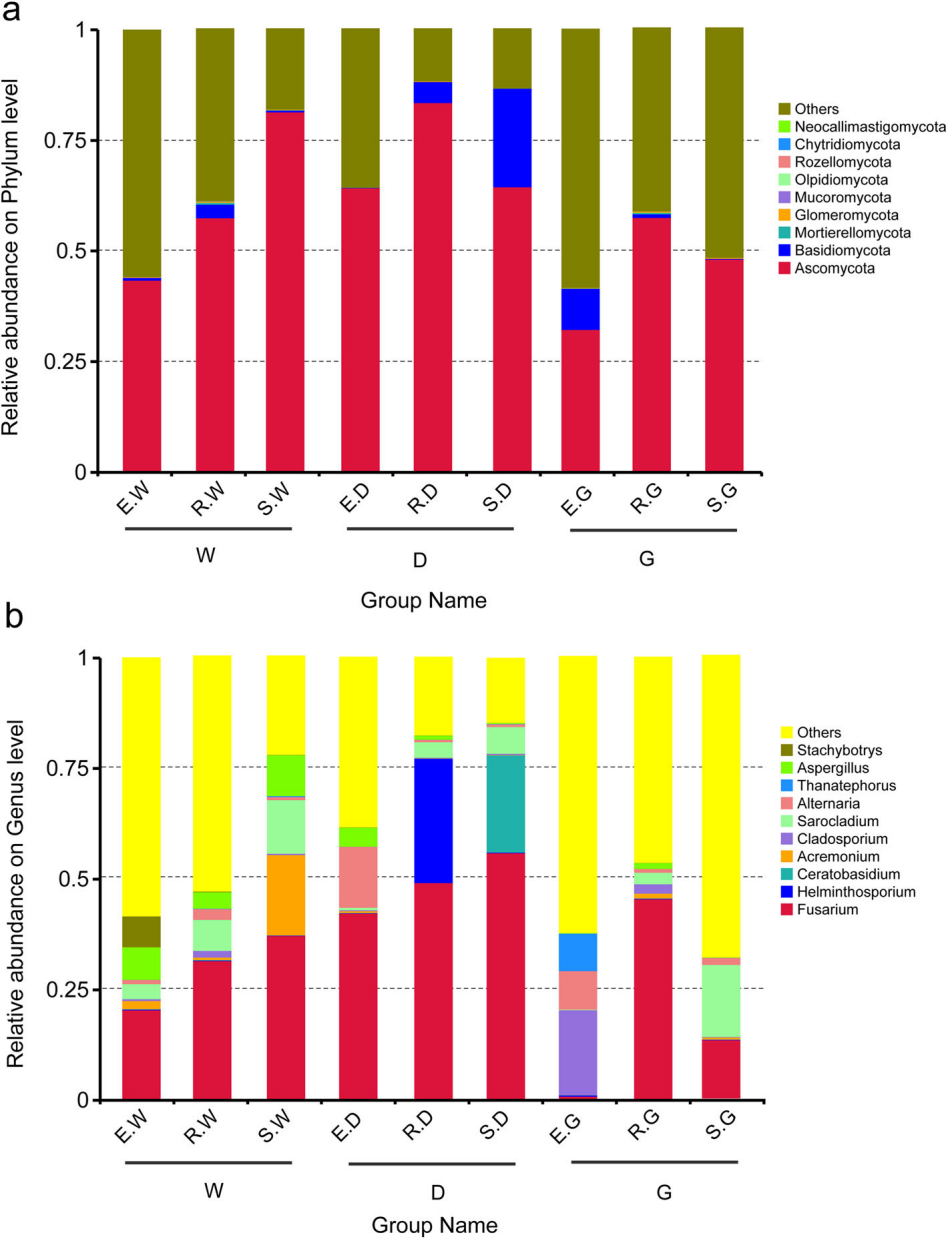
Histograms of relative abundance of the top 10 endophytic fungi at the phylum (**a**) level of taxonomy and at the genera (**b**) level of taxonomy. Description: (**a**) and (**b**) Ordinate both are the relative abundance of species, others mean less or not annotated; abscissa is the group name that has identical meanings as described in Fig. 2

Ascomycota phylum was more abundant than other fungal phyla. It accounted for 43.608, 57.392, 81.344, 64.176, 83.474, 64.392, 32.231, 57.237, and 47.782% of the total number of species in E. W, R. W, S. W, E. D, R. D, S. D, E. G, R. G, and S. G samples, respectively (Fig. 5a). With the increasing growth period, the relative abundance of Ascomycota in the *G. uralensis* roots increased significantly. Besides, Basidiomycota was the predominant phylum in R. D (4.689%), S. D (22.261%), and EG (9.331%) samples. The growth period significantly increased the relative abundance of Basidiomycota in *G. inflata* root, but Basidiomycota’s relative abundance was significantly reduced in *G. glabra* root (Fig. 5a).

The *Fusarium* genus was more abundant than other genera in all the samples. The individual groups ranged from 56.12% (S.D) to 0.666% (E.G) (Fig. 5b). The relative abundance of the *Fusarium* genus increased significantly with the growth period in the *G. uralensis* and *G. inflata* roots. Meanwhile, with the increasing growth period, the relative abundance of *Sarocladium* in the three medicinal licorice species also increased. However, *Cladosporium’s* relative abundance in *G. glabra* reduced significantly with the growth period (Fig. 5b).

**Fig. 6:**
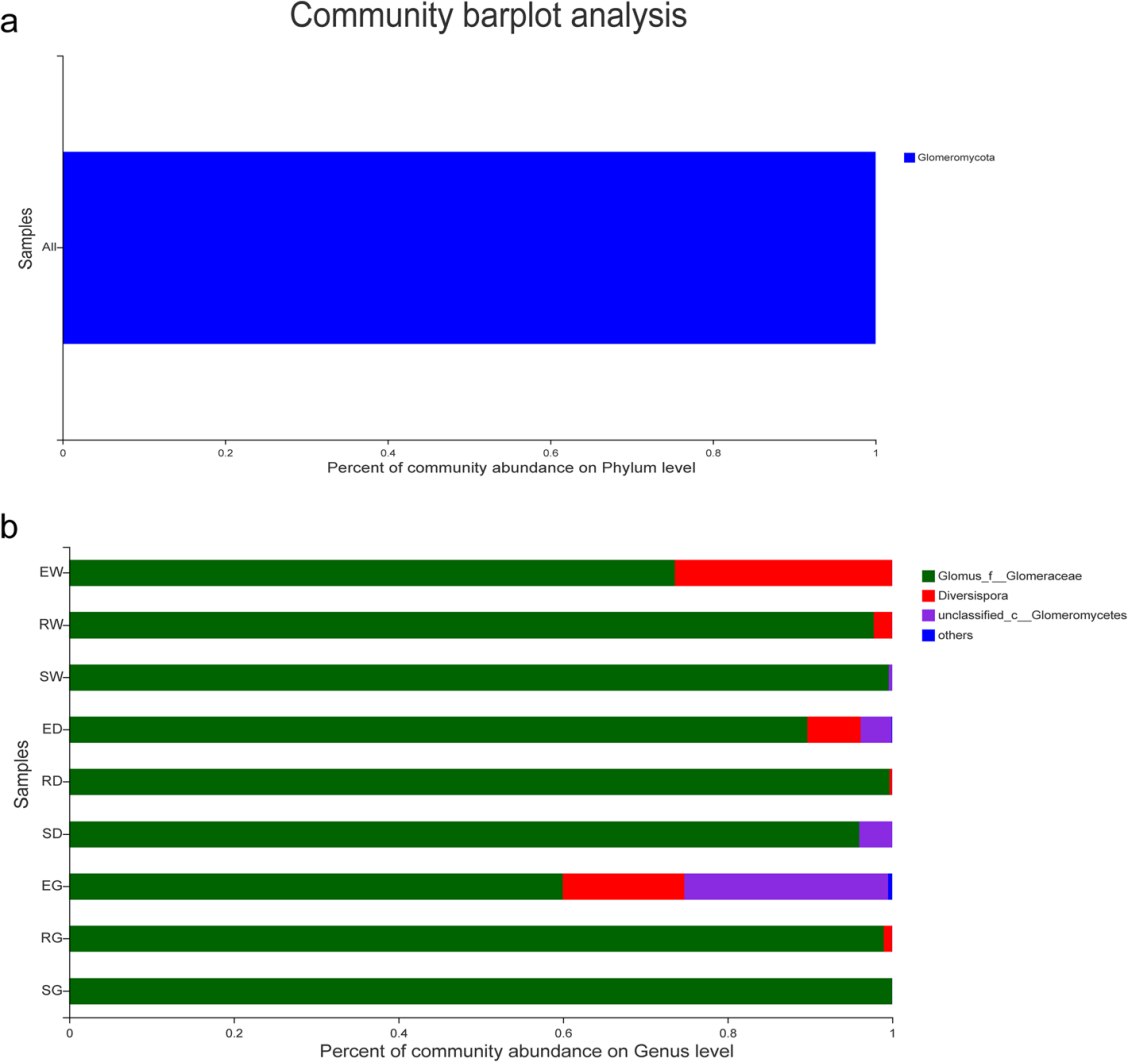
Histograms of relative abundance of the top 10 arbuscular mycorrhizal fungi phylum (**a**) and genera (**b**). Description: Abscissa is the relative abundance of species; others mean less or not annotated; Ordinate is the group name that has identical meanings as described in Fig. 2

AMF communities’ OTU were taxonomically annotated into three distinct genera. *Glomus* genus was the dominant genus in all the samples. It accounted for 73.595, 97.771, 99.594, 89.690, 99.685, 96.015, 59.967, 98.980, and 99.998% of the total number of species in E. W, R. W, S. W, E. D, R. D, S. D, E. G, R. G, and S. G, respectively. *Diversispora* genus was the most dominant genus in E. W (26.394%), E. G (14.792%), and E. D (6.479%) samples (Fig. 6b). The relative abundance of *Glomus* increased significantly with the increasing growth period; however, the relative abundance of *Diversispora* was reduced significantly (Fig. 6b).

**Fig. 7:**
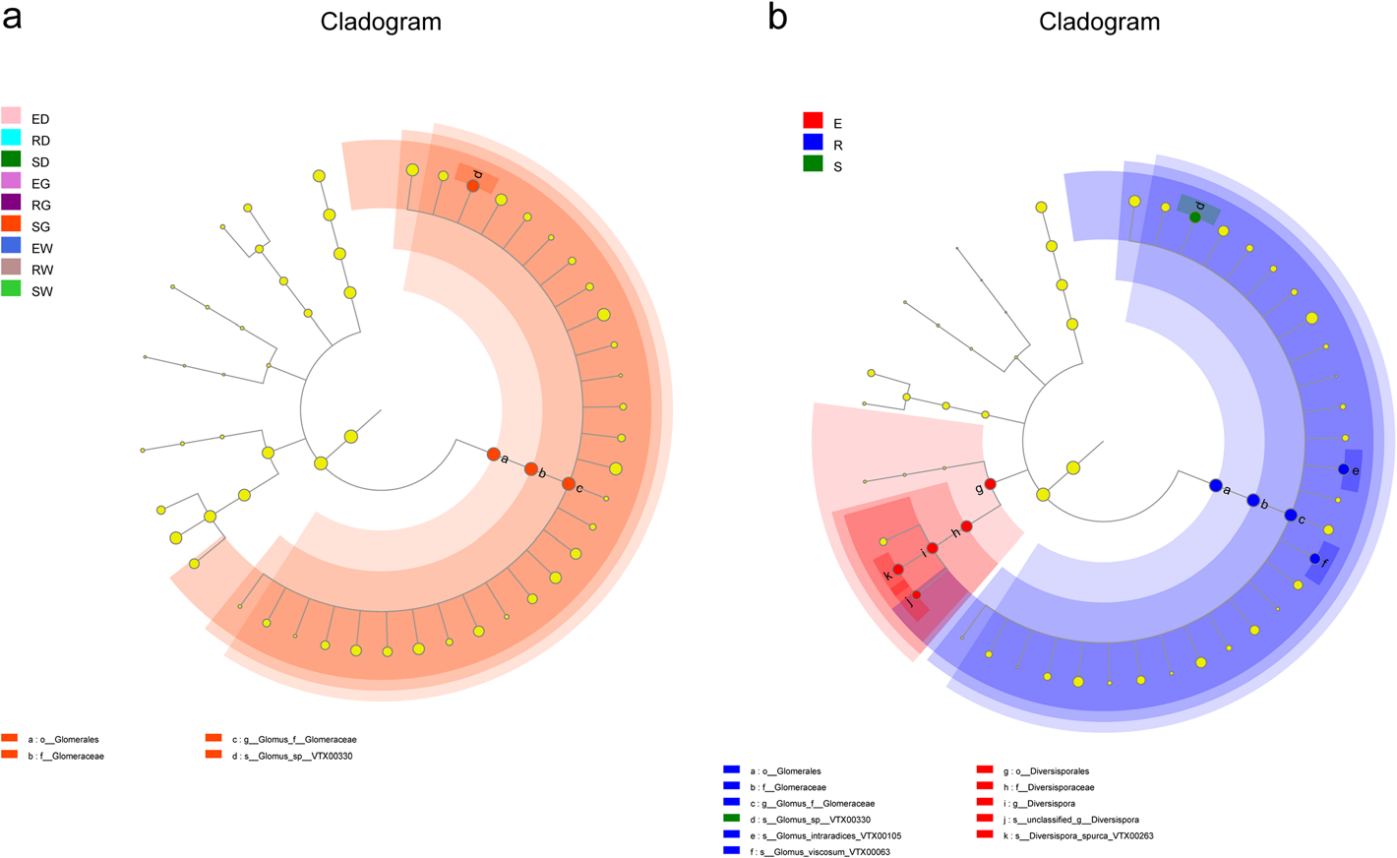
Linear discriminant analysis effect size (Lefse) analysis of differences in AMF community composition as a function of growth period. Description: In cladograms, the circle radiating from inside to outside represents the taxonomic level from the Phylum to the species. Each small circle at a different taxonomic level represents microbial groups that were significantly enriched in the corresponding groups and that significantly influenced the differences between groups, and the diameter of the small circle is proportionate to the relative abundance of species. Light yellow small circle represents microbial groups with no significant differences. Group name that has identical meanings as described in Fig. 2

LEFSe (LDA Effect Size) analysis was employed to discern the statistically significant differences in the relative abundance of soil’s AMF communities and validate the correlation of soil AMF communities during their growth period. In each group, only S. G samples showed significant enrichment of three biomarkers for three species: *Glomerales*, *Glomeraceae*, and *Glomus* (Fig. 7a). Concerning the growth period, a total of 11 biomarkers were employed to discern significant differences in the AMF community. As per the outcomes of this analysis, significant differences in abundance were observed in the 3rd year (1 Taxon: *Glomus-sp.-VTX00330*), 1st year (5 Taxa: Diversisporaceae, Diversisporales, *Diversispora*, *Diversispora-spurca-VTX00263* and unclassified _ *Diversispora*), and 2nd year (5 Taxa: Glomerales, Glomeraceae, *Glomeraceae, Glomus-intraradices-VTX00105*, *Glomus-viscosum-VTX00063*) of the growth period (Fig. 7b).

### Relationship of root’s secondary metabolites with soil’s physicochemical properties and microbial community

**Fig. 8:**
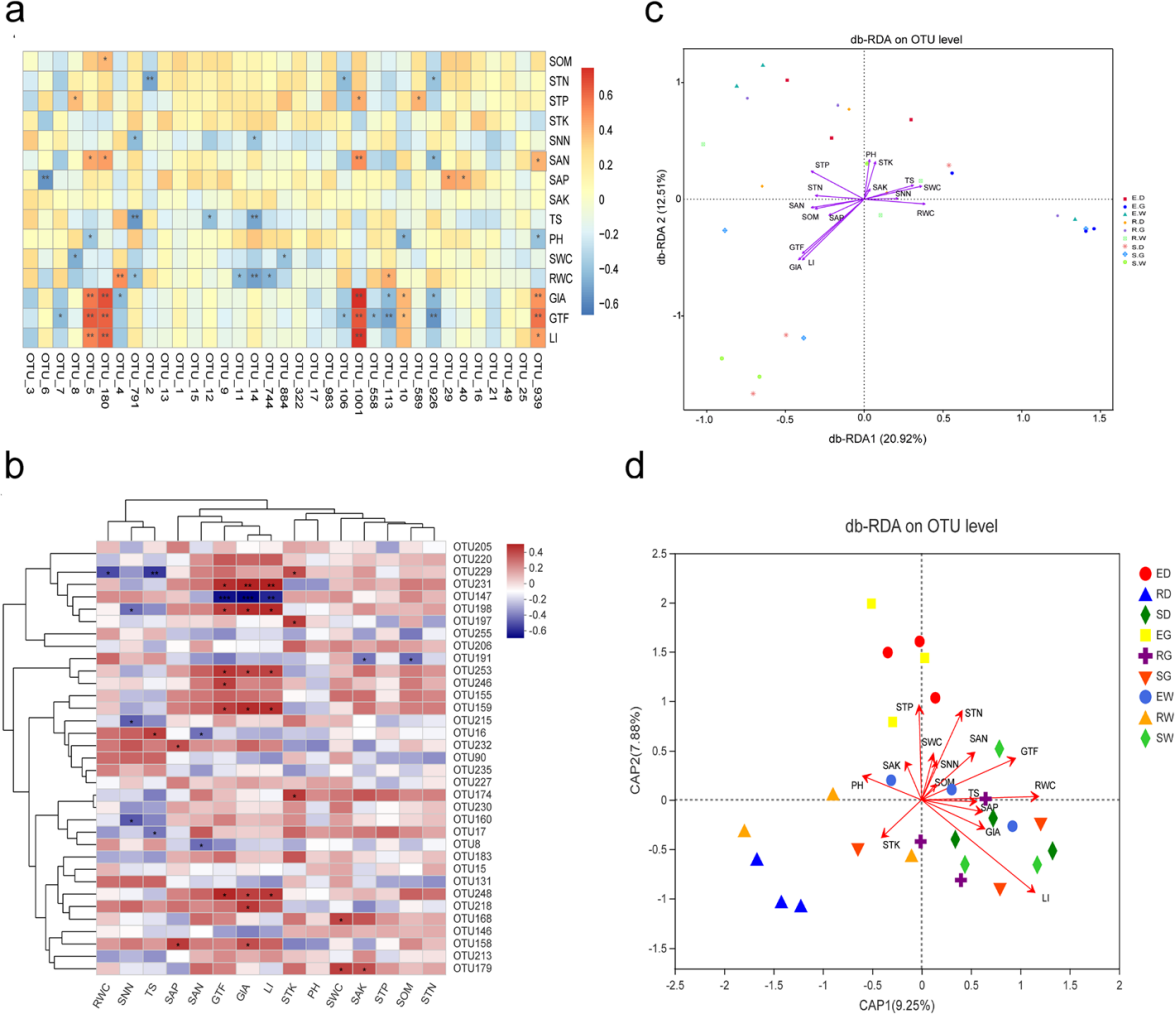
Relationship between secondary metabolites and soil physical and chemical properties and microbial community. Description: Heat maps of spearman correlation analysis between the top 20 OTUs, secondary metabolite and soil properties with (**a**) corresponding to Endophytic fungal community and (**c**) corresponding to AMF community, respectively. The mark * is significance test *p* < 0.05. Db-RDA analysis based on OTU levels that mainly used to reflect the relationship between microorganisms and environmental factors. Endophytic fungal community and AMF community corresponding to (**b**) (**d**), respectively

The correlation between the top 35 OTUs of endophytic fungal community, secondary metabolites, and soil’s physicochemical properties is depicted using Spearman heat map (Fig. 8a). GIA, GTF, and LI content were significantly and negatively correlated to OTU-5, OTU-180, OTU-1001, and OTU-939 (*P* < 0.05), in line with the SAN content in the soil (Fig. 8a). OTU-180 and OTU-1001 belonged to the genus *Fusarium*, Ascomycota phylum. OTU-5 belonged to the genus *Sarocladium*, Ascomycota phylum. Out of the top 35 OTUs of the AMF community, the contents of GTF, GIA, and LI were significantly and positively correlated to OTU-248, OTU-159, OTU-253, OTU-198, and OTU-231 (R > 0, *P* < 0.05), which belonged to genus *Glomus*, but negatively correlated to OTU-147 (R < 0, *P* < 0.05), which belonged to genus *Diversispora* (Fig. 8b).

The distance-based redundancy analysis (db-RDA) based on Bray-Curtis distance revealed that GIA (*r*^*2*^ = 0.434, *P* < 0.01), GTF (*r*^*2*^ = 0.363, *P* < 0.01), and LI (*r*^*2*^ = 0.417, *P* < 0.01) were crucial environmental driving factors, which influenced the distribution of endophytic fungal communities, resulting in 33.43% of the overall variability in the composition of endophytic fungal communities (Fig. 8c). In AMF community, LI (*r*^*2*^ = 0.477; *P* < 0.01), RWC (*r*^*2*^ = 0.300; *P* < 0.05) were the crucial environmental driving factors, which affected the distribution of AMF community (Fig. 8d). It suggests that endophytic fungal communities are more sensitive to secondary metabolites than AMF communities. Additionally, as per the Mantel test, GIA, GTF, and LI were the environmental factors with the highest correlation to endophytic fungal communities (r = 0.273, *P* = 0.014) (Table 3). Environmental factors, i.e., RWC, GIA, GTF, and LI, were highly correlated to the AMF communities (r = 0.358, *P* = 0.003).

**Table 3:** Mantel test on the correlations between the relative abundance of OTUs and soil or root variables

Variable	Endophytic Fungi		Soil AMF	
	***r***	***P*** value	***r***	***P*** value
SOM + STN + STP + STK + SNN + SAN + SAP+SAK + TS + PH + SWC	0.119	0.158	0.038	0.785
RWC + GlA + GTF + LI	0.209	0.024	0.358	0.003
STN + STP + STK	0.075	0.776	0.063	0.533
SOM + SNN + SAN + SAP+SAK + TS + PH + SWC	0.178	0.075	0.038	0.806
GlA + GTF + LI	0.273	0.014	0.110	0.200
GlA + GTF + LI + STN + STP + STK	0.007	0.522	0.065	0.634
GlA + GTF + LI + SOM + SNN + SAN + SAP+SAK + TS + PH + SWC	0.168	0.080	0.046	0.736

Description: Variable is the information of environmental factors. *r* is the correlation coefficient, the larger *r* value is, the greater the correlation between environmental factors and species abundance information is. *P* value is the *p*-value of significance test. *P* < 0.05 indicates statistical significance

## Discussion

In this study, levels of secondary metabolites, i.e., GIA, LI, and GTF, in the three *Glycyrrhiza* species roots increased significantly with the growth period (Fig. 1), in line with the study by Yang et al. [27]. In medical licorice plants, bioactive compounds, the end-product of physiological activities [28], maintain their concentration during the growth period and protect plants from neighboring plants, pathogens and changing environmental conditions [29]. Rhizospheric microbiota influences the production of secondary metabolites in licorice root. In this study, multiple arbuscular mycorrhizal fungal species were present in the rhizospheric soil (Fig. 6), including *Glomus*. In a study by Chen et al. [13], *Glomus mosseae* inoculation improved the root structure, photosynthetic efficiency, and flavonoid accumulation in *G. uralensis*. Xie et al. [1] demonstrated that the symbiotic activity of AMF increased phosphorus absorption and the accumulation of glycyrrhizin and liquiritin in the *G. uralensis* plants. Also, glycyrrhizic acid, liquiritin, and flavonoids were significantly and positively correlated to genus *Glomus* (Fig. 8b). Thus, the current study demonstrated that AMF colonization effectively increased the production and accumulation of secondary metabolites in the roots of three medicinal licorice species.

As per the outcomes of the current study, the contents of GIA, GTF, and LI in medicinal licorice roots were significantly and negatively correlated to TS content (Table 2 and Fig. S1) of soil. It increased the osmotic and ionic stress in plants, a major abiotic limiting factor for medicinal licorice plants’ growth and development in the arid and semi-arid regions [30]. It can be attributed to the higher concentration of salts (primarily Na^+^) in the soil, reduced uptake of water and nutrients by plant, and photosynthesis rate [31]. It restricted the nutrient content required for the growth and development of plants [32]. Interestingly, soil’s TS content was significantly reduced during the medicinal licorice’s growth period (Table 1). It indicated that licorice plants in arid areas could effectively alleviate the abiotic stress due to soil’s salt content in arid and semi-arid areas and produce the bioactive compound in the roots. Wang [33] and Zahra [34] et al. demonstrated that appropriate salt stress increased the accumulation of effective components, i.e., triterpenoid, flavonoids, and glycyrrhizin in licorice root through overexpression of gene or proteins involved in biosynthetic pathways.

As per previous studies, the level of nutrients in the soil decreased during perennial growth and development of plants due to plant’s nutrient uptake [35, 36]. In line with these reports, this study showed the plant growth significantly reduced the TS, SNN, available potassium (SAK), and STK contents in soil (Table 1). Conversely, the content of total nitrogen (STN), total phosphorus (STP), STK, organic matter (SOM), SAN, and SAK in the soil during the 2nd year of growth period was slightly higher than 1st and 3rd year of the growth period. It might be correlated to secondary metabolite production in medicinal plant’s roots, leaf litter, and altered climatic conditions, including rainfall regimen and temperature.

Firstly, litterfall links the transport of nutritional ingredients from vegetation to soil’s ecosystem. As per previous studies, certain dissolved organic compounds, such as phenolic compounds and terpenoids, leached from leaf litter increased soil’s microbial activity and mineralization of soil nutrients, including organic nitrogen and organophosphorus, through the metabolism of enzymes [37, 38]. Thus, the quality and quantity of litterfall influence the nutrient cycling, retention and erosion of nutrients, and microbial respiration rate in the soil [39]. Secondly, climatic condition, particularly rainfall, increases the dissolution of organic matter, including proteins, phenols, and carbohydrates, and inorganic nutrients from plant’s leaf litter [40]. In this study, changes in nutrient levels (SOM, STN, STP, STK, SAN, and SAK) were in line with the rainfall during the experimental growth period of three years. Rainfall in the 2nd year (65 mm) was higher than rainfall in the 1st year (46.9 mm) and 3rd year (23.2 mm), which paralleled the levels of SOM, STN, STP, STK, SAN, and SAK. It showed that the differences in annual rainfall might alter the soil’s ecological environment. Thus, it suggested that climatic conditions might positively influence growth, development, and effective component of medicinal licorices in the near future.

Furthermore, the richness of endophytic fungi in the 2nd year was slightly higher than in the 1st and 3rd year (Fig. 2). We speculated that a higher rainfall increased the dissolution of organic matter in the soil, the primary source of nutrients and energy for microbes, resulting in increased soil fertility and microbial activation [41].

Dominant microbial communities play a crucial role in soil nutrient cycling and secondary metabolite production in roots [42]. In this study, HTS was used to discern the composition and diversity of endophytic fungal and AMF communities and elucidate the role of secondary metabolites on fungal community composition in medicinal licorice’s roots and rhizospheric soil. As per the outcomes of the current study, the composition and diversity of the endophytic fungal and AMF communities were altered during the licorice’s growth period (Fig. 4). These findings were in line with the study by Zhang et al. [43], which demonstrated that the microbial migration in rice plant’s root microflora was correlated to the growth period and developmental stages. The diversity of the endophytic fungal community was influenced by both growth period and species (Fig. 3), whereas the AMF community was affected more by the growth period. It indicated that soil and plant interaction affected endophytic fungal and the rhizospheric AMF communities differently, evident by the temporal succession of root’s endophytic fungal communities and the simultaneous changes in root’s secondary metabolites during the growth period of three licorice species. Endophytic fungi are present in host plant root’s as a microbiome. The qualitative composition of root exudates depends on plant species, plant developmental stages, and other environmental factors [44]. It directly influences the composition of endophytic fungi. The interaction between plants and microbes is highly dynamic and driven by a deterministic selection of environmental factors based on coevolutionary pressures [45, 46]. Multiple studies have demonstrated that plants influence the microbial community through specific root exudates. In a study by Zhou et al. [47], coumaric acid was applied to cucumber seedlings to increase the abundance and alter bacterial and fungal communities’ composition. Carvalhais et al. [48] demonstrated that activation of induced systemic resistance in plants, for instance, induction of a jasmonic acid defense pathway, significantly altered the rhizospheric microbial community.

Furthermore, the application of exogenous substances for a long time affected AMF community composition in the *Glycyrrhiza’s* rhizospheric soil. The soil acts as a natural habitat for the rhizospheric AMF community. As shown in the previous study, altered nutrient content in the soil triggered the shift in AMF’s mycelium density and spore density for the resource allocation to the active and static structure of soil’s internal environment [49]. Previous studies have shown that a continuous increase in soil nutrient content, for instance, P, increased the AMF spore’s density [50]. Min Sheng et al. [51] demonstrated that prolonged application of phosphate fertilizer in soil indirectly increased the AMF spore’s and mycelium density in the top layer of the soil. Besides, as per other studies, organic matter in the soil served as the primary substrate and energy resource for microbes [52]. As biological nutrients, certain AMF species can directly consume simple organic matter from different resources for growth and reproduction [53].

With plants growing, the licorice plants selected for specific microbial communities composition, including both endophytic fungi (Ascomycota, Basidiomycota, Mortierellomycota and Glomeromycota) (Fig. 5), and AMF (*Glomus*, *Diversispora*) (Fig. 6). Due to its adaptability, the Ascomycota phylum is abundant in diverse ecosystems, including terrestrial and coastal marine habitats [54]. It might interfere with the food chain of the soil. Moreover, multiple studies have [55, 56] indicated that Ascomycota plays a crucial role in increasing nutrient and SOMdecomposition in the soil. Besides, it is a dominant phylum and participates predominantly in the assimilation of root secretion and SOM degradation in the host plant. Multiple studies [57, 58] have shown that the Glomeromycota phylum has a special mutualistic symbiotic relationship with the roots of more than 80% of terrestrial plants (for instance, arbuscular mycorrhiza). Its hyphae links the plant’s roots and soil nutrient reserves [49] and enhances the solubility and availability of multiple nutrients, improves the soil structure, and provides a protective barrier to plants for micronutrient absorption [59, 60]. It increases the nutrient absorption by the licorice plant roots and promotes the growth of licorice plants. Besides, Glomeromycota is more resistant to environmental disturbances and pressures and thus plays a crucial role in fulfilling ecological functions [61]. In the current study, the fungi discussed above were present in all the samples. Thus, we speculated that these fungi are closely co-related to the growth of the licorice plant. An in-depth analysis of the interaction between these fungi and licorice might provide crucial data for licorice cultivation.

A complex interaction between physicochemical and biological components of the rhizosphere and its surrounding environment (soil and climate) results in highly structured microbial communities [62, 63]. As per the current study, secondary metabolites (GIA, GTF, and LI) serves as the crucial driving factors in the composition and distribution of AMF and endophytic fungal communities of three medicinal licorice species (Fig. 8 and Table 3), in line with the study by Li et al. [64]. Phytochemicals secreted by plant roots mediate multiple interactions, such as plant-microbe, plant-plant, and plant-fungus interactions. A plethora of studies [65] has shown that secondary metabolites in root exudates are highly dependent carbon substrates of microbial colonization. It plays a crucial role in the interaction between plants and microbes. It is a well-known fact that flavonoids stimulate or inhibit rhizobial nod gene expression and chemically attract the roots [66]. Flavonoids can also act as a signaling component and establish arbuscular mycorrhizal symbiosis, stimulate mycorrhizal spore germination and mycelium branch, and mediate allelopathy between plants [67]. However, the underlying mechanism for signaling between exudates-mycorrhiza-fungi requires an in-depth analysis. The current study provides reference data to decipher the roles of root exudates in promoting these beneficial multi-party interactions.

## Conclusions

This study investigated the temporal succession of root-related endophytic fungal and AMF communities in the licorice plant as well as altered soil characteristics and secondary metabolites in the root at different time-points. The diversity and richness of endophytic fungal communities were significantly affected by the growth period and species as compared to the AMF community. However, endophytic and AMF community composition was influenced by the growth period. During the plant’s growth period, the contents of three secondary metabolites in roots increased per year, and the soil’s physicochemical properties were altered, which contributed to the overall differences in composition and distribution of endophytic fungal and AMF communities. As per the outcomes of this study, the endophytic fungal communities were more sensitive to secondary metabolites than AMF communities. To characterize the role of these fungi in the secondary metabolite accumulation, an in-depth analysis is required. The current study provides novel insights into the interaction between rhizospheric microbes and root exudates.

## Methods

### Soil and root sampling

Field experiments were conducted during August 2017, August 2018, and August 2019 at the licorice planting base of South Yanqi (86°17′E, 42°11′ N), Xinjiang Province, China. The study area was located at an altitude of 1073.3 m with a temperate continental desert climate and sandy soil. The average annual temperature during the study period (2017, 2018, and 2019) was 10.06 °C, 8.92 °C, and 9.98 °C, respectively. The average annual rainfall during 2017, 2018, and 2019 was 46.9 mm, 65 mm, and 23.2 mm, respectively. The licorice seeds used in the experiments were procured from Xingjiang beiling licorice Technology Co., Ltd. (Xinjiang, China), and seeds were covered with a thin soil layer and wrapped in plastic. The plastic film was removed following seed germination. The licorice seedlings were transplanted from the nursery to the field and watered during germination, middle, and maturity stages of the growth period.

Roots and rhizospheric soil were sampled during August 2017, August 2018, and August 2019 from three licorice species (*G. inflata*, *G. uralensis,* and *G. glabra*), and staggered sampling was practiced to maintain sample uniformity. In this study, samples were collected from 9 (3 species × 3 duplicates) plots (4 m × 4 m). Three homogenous composite samples were collected from each site, and each sample consisted of five randomly selected roots of well-grown licorice plants. Five well-grown licorice plants were randomly selected for each sample plot based on “Z” type to dig out intact plant roots. To separate loose bulk soil attached to the roots and the rhizospheric soil samples (0–40 cm depth), roots were shaken several times without damaging the root structure. Five soil samples were mixed to obtain a uniform composite soil, which was further divided into 2 sub-samples; one was stored in liquid nitrogen for DNA extraction and another part for analyzing physiochemical properties. Sterile scissors were used to cut down the roots (0–40 cm). Each root sample was equally divided into two parts, where one part was used to determine the content of secondary metabolites. Another was placed into sterile plastic bags and immediately taken to the laboratory in an icebox for disinfection and sterilization of the root surface, as described previously [68], and stored in liquid nitrogen until DNA extraction. A total of 54 experimental samples were obtained, including 27 soil samples and 27 root samples (3 years × 3 species × 3 duplicates), until further processing.

### Determination of physicochemical characteristics and secondary metabolites in licorice plant’s roots

The rhizospheric soil samples were naturally air-dried in the laboratory to attain constant weight so that it could pass through 2 mm sieve before determining the physicochemical properties of soil. Soil pH was determined through the conventional method using a pH meter and soil suspension (1:5 water and soil ratio). Soil moisture content (SWC) was measured through gravimetric analysis. As per the previous method [68], perchloric acid (sulfuric acid digestion method) with FOSS 1035 automatic nitrogen analyzer was used to determine the concentration of STN in the soil; STP in the soil was determined using the molybdenum antimony anti-colorimetric method and Agilent CARY60 UV spectrophotometer; STK in the soil was determined using the acid solution and atomic absorption spectrometry; SOM was determined by external heating method; The TS was determined using atomic absorption spectrometry. The concentration of ammonium nitrogen (SAN) and SNN in soil was determined using the 0.01 M calcium chloride extraction method, as described previously by Bao et al. [69]. The available phosphorus (SAP) was determined through the sodium bicarbonate extraction (molybdenum-antimony anti-colorimetry) method, as described previously by Bao et al. [69]. The SAK was estimated using the ammonium acetate extraction method and atomic absorption spectrometer.

The licorice root samples were dried to constant weight in the laboratory oven (60 °C/72 h) before passing through the 60 mesh sieve, and samples were ground to powder using pestle and mortar [1]. At room temperature, 0.2 g of licorice root powder samples were extracted using an ultrasonic bath (250 W, 40 KHz) and 71% chromatographic methanol. The resulting solution was centrifuged at 12,000 rpm for 10 min, and the supernatant was filtered using 0.22 μm pore size membrane (Agilent, USA). As described previously [68], two representative secondary metabolites, i.e., LI and GIA in licorice root was determined using high-performance liquid chromatography (HPLC) on Agilent ZORBAX SB-C18 column (150 mm × 4.6 mm). Details of the GIA and LI data are presented in Fig. S2 and Fig. S3, respectively. The content of GTF was determined using an ultraviolet spectrophotometer (334 nm), and glycyrrhiza standard (CAS # 551–15-5) from Solarbio was used as a reference.

### DNA extraction

The total DNA was isolated from 0.5 g of soil and root samples using FastDNA® Spin Kit for Soil (MP Biomedicals, USA) and DNA Quick Plant System Kit (Tiangen, China), as per the manufacturer’s instructions. DNA concentration and purity were determined using NanoDrop2000 (Thermo Fisher Scientific, USA), and the DNA integrity of isolated DNA was visually inspected using 1% agarose gel electrophoresis (Fig. S4). The total DNA was diluted to 1 ng/μL using sterile water as a PCR reaction template.

### PCR amplification of root samples

DNA samples obtained from root samples were PCR amplified using specific primers. To amplify ITS genes of ITS1-5F region, specific primers: 1737F (5′-GGAAGTAAAAGTCGTAACAAGG-3′) and 2043R (5′-GCTGCGTTCTTCATCGATGC-3′), containing barcodes were employed [70]. Additionally, to ensure efficiency and accuracy, PCR amplification was performed using Phusion® High-Fidelity PCR Master Mix (New England Biolabs), and reactions were carried out in the thermocycler PCR system (ABI GeneAmp®9700, ABI, USA) under the following thermal cycling program: 95 °C/3 min (initial denaturation) followed by 32 cycles of 95 °C/30 s, 52 °C/30 s, 72 °C/30 s, and lastly at 72 °C/5 min (final extension).

### PCR amplification of soil samples

The AMF genes of the AMV4-5NF_AMDGR region were amplified using nested PCR and specific primers. The first round of PCR amplification was carried out using specific AMF primers: AML1F (5′-ATCAACTTTCGATGGTAGGATAGA-3′) and AML2R (5′-GAACCCAAACACTTTGGTTTCC-3′) with the following thermal cycling program: 95 °C/3 min (initial denaturation) followed by 32 cycles of 95 °C/30 s, 55 °C/30 s, 72 °C/45 s, and lastly at 72 °C/10 min (final extension). In the second round of the PCR amplification step, PCR products from the first round were used as the template using specific primers: AMV4-5NF (5′-AAGCTCGTAGTTGAATTTCG-3′) and AMDGR (5′-CCCAACTATCCCTATTAATCAT-3′) containing barcodes. The nested PCR amplification was performed with the following condition: 95 °C/3 min (initial denaturation) followed by 30 cycles of 95 °C/30 s, 55 °C/30 s, 72 °C/45 s, and lastly at 72 °C/10 min (final extension). Both the round of PCR amplification were performed in triplicate, and each reaction contained 20 μL reaction mixture, which included 4 μL of 5X FastPfu Buffer, 2 μL of 2.5 mM dNTPs, 0.4 μL of forward/reverse primer (10 μM), 0.4 μL of FastPfu Polymerase, 0.2 μL of BSA, 1 μL template DNA, and 12 μL ddH_2_O was added to make up the volume to 20 μL.

### PCR products purification and Illumina HiSeq sequencing

PCR products were mixed with an equal volume of 1X loading buffer and detected using 2% agarose gel electrophoresis. Target strips were cut and purified using the AxyPrep DNA Gel Extraction Kit (Axygen Biosciences, Axygen, USA) and quantified using Quantus™ Fluorometer (Promega, USA), as per manufacturer’s standard. The cDNA library was constructed using the NEXTFLEX Rapid DNA-Seq Kit (Bioo Scientific, USA), as per the manufacturer’s instruction. The quality of the library was evaluated using Qubit® 2.0 Fluorometer (Thermo Scientific) and Agilent Bioanalyzer 2100 system. This cDNA library was sequenced by the Beijing Compass Biotechnology Co., Ltd. (Beijing, China) on Illumina HiSeq2500 platforms.

### Bioinformatics analysis and statistical analysis

The paired-end reads from sequencing were assigned to respective samples using Cutadapt software [71], based on their specificity. Barcode and primer sequences were truncated from these reads. Flash software version 1.2.7 [72] was employed to cut and splice the remaining paired-end reads of each sample, and these reads were assembled to obtain the original label. High-quality clean data was obtained by filtering the original data under specific filtering conditions [73, 74]. Additionally, to segregate chimeric sequences and to eliminate the non-microbial reads, for instance, chloroplast and mitochondrial reads, the reads were compared with the Unite database (https://unite.ut.ee/) [75] using UCHIME [76]. Thus, clean reads were generated. UPARSE software version 7.0.1001 (http://drive5.com/uparse/) [77] was used to cluster the sequences into the same operational taxonomic units (OTUs) with ≥97% similarity, and representative sequence with the highest frequency was selected for further annotation. The Unite database was used to execute annotated information for each representative sequence. MUSCLE software version 3.8.31 (http://www.drive5.com/muscle/) [78] was used to align multiple sequences, analyze the phylogenetic relationship of different OTUs and the differences between the dominant microbial community species in different groups. OTUs abundance was normalized using the samples containing the least number of sequences. α and β diversity were analyzed based on the normalized data.

In this study, the alpha diversity index, including Chao1, ACE, Shannon, Simpson, and commodity coverage of the samples was calculated using QIIME version 1.7.0 and represented using R software version 2.15.3. The average values of each group were used to construct species histograms of phylum and relative abundance of the genus. R software version 2.15.3 was employed to perform the PCoA, Wilcoxon rank-sum test based on weighted unifrac, Spearman correlation between species and abiotic factors, db-RDA, NMDS, Mantel tests, and Venn diagram. The linear discriminant analysis (LDA) was performed using LEfSe software, and LDA was used with the default filter value of LDA score 4 to assess differences between statistical groups. The statistical analysis of variance (ANOVA) and Spearman correlation analysis was performed using SPSS version 19.0 (IBM Inc., Armonk, USA), with the significance level set to 0.05. Pearson correlation analysis was performed for soil’s secondary metabolites and physicochemical properties.

## Supplementary Information

**Additional file 1.**

## Data Availability

All data generated or analysed during this study are included in this published article and its supplementary information files.

## Abbreviations

HPLC: high-performance liquid chromatography
AMF: arbuscular mycorrhizal fungi
HTS: High-throughput sequencing
LI: liquiritin
GTF: total flavonoids
GIA: glycyrrhizic acid
NMDS: Non-metric multidimensional scaling
PCoA: principal coordinate analysis
LDA: linear discriminant analysis
db-RDA: distance-based redundancy analysis
OTUs: operational taxonomic units
SWC: Soil moisture content
STP: Total phosphorus
SOM: organic matter
STN: total nitrogen
STK: total potassium
TS: total salt
SNN: nitrate nitrogen
SAN: ammonium nitrogen
SAP: available phosphorus
SAK: available potassium
