# Supplementary material for: Succession of endophytic fungi and arbuscular mycorrhizal fungi associated with the growth of plant and their correlation with secondary metabolites in the roots of plants
Source: BMC Plant Biol. 2021 Apr 5;21:165. doi: 10.1186/s12870-021-02942-6 (PMC8022407; doi:10.1186/s12870-021-02942-6)
Supplement: Supplementary file 1 — Additional file 1. [file 12870_2021_2942_MOESM1_ESM.docx]

Succession of endophytic fungi and arbuscular mycorrhizal fungi associated with the growth of plant and their correlation with secondary metabolites in the roots of plants

**Hanli Dang ^1^, Tao Zhang ^1^, Zhongke Wang ^1^, Guifang Li ^1^, Wenqin Zhao^1^, Xinhua Lv^1^, and Li Zhuang ^1^***

^1^ College of life Sciences, Shihezi University, Shihezi City, 832003, Xinjiang, China

* Correspondence and requests for materials should be addressed to Li Zhuang (E-mail: 3033573705@qq.com).

# Additional file

## Figure S1. Stepwise multiple linear regression model


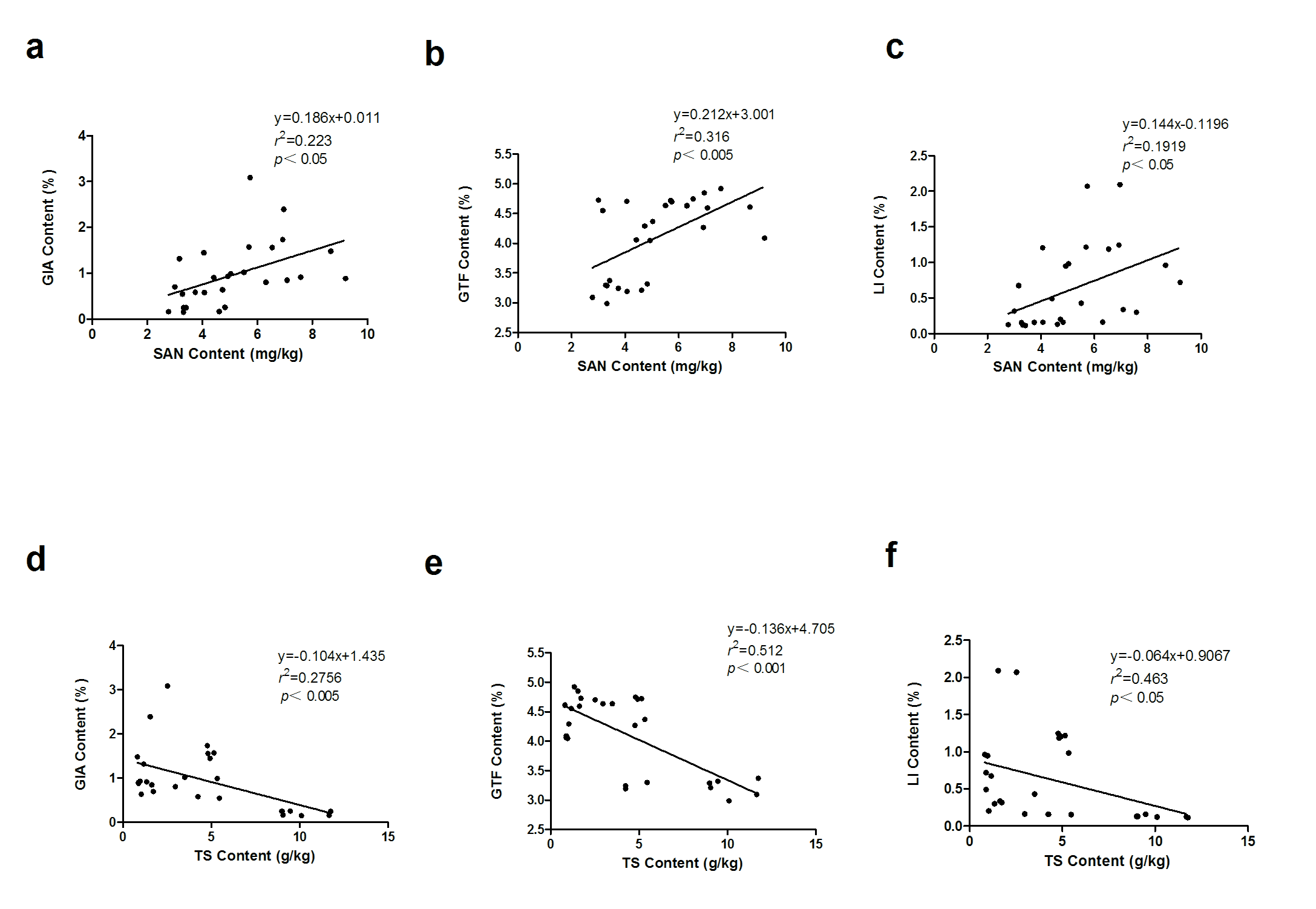


**Figure S1** Stepwise multiple linear regression model

Description: The content of soil ammonium nitrogen (SAN) (a, b, c) and total salt (TS) (d, e, f) were used as independent variables, and the content of root secondary metabolites (GIA, GTF and LI) were used as dependent variables, respectively.

## Figure S2. glycyrrhizic acid content in the root of three licorices species


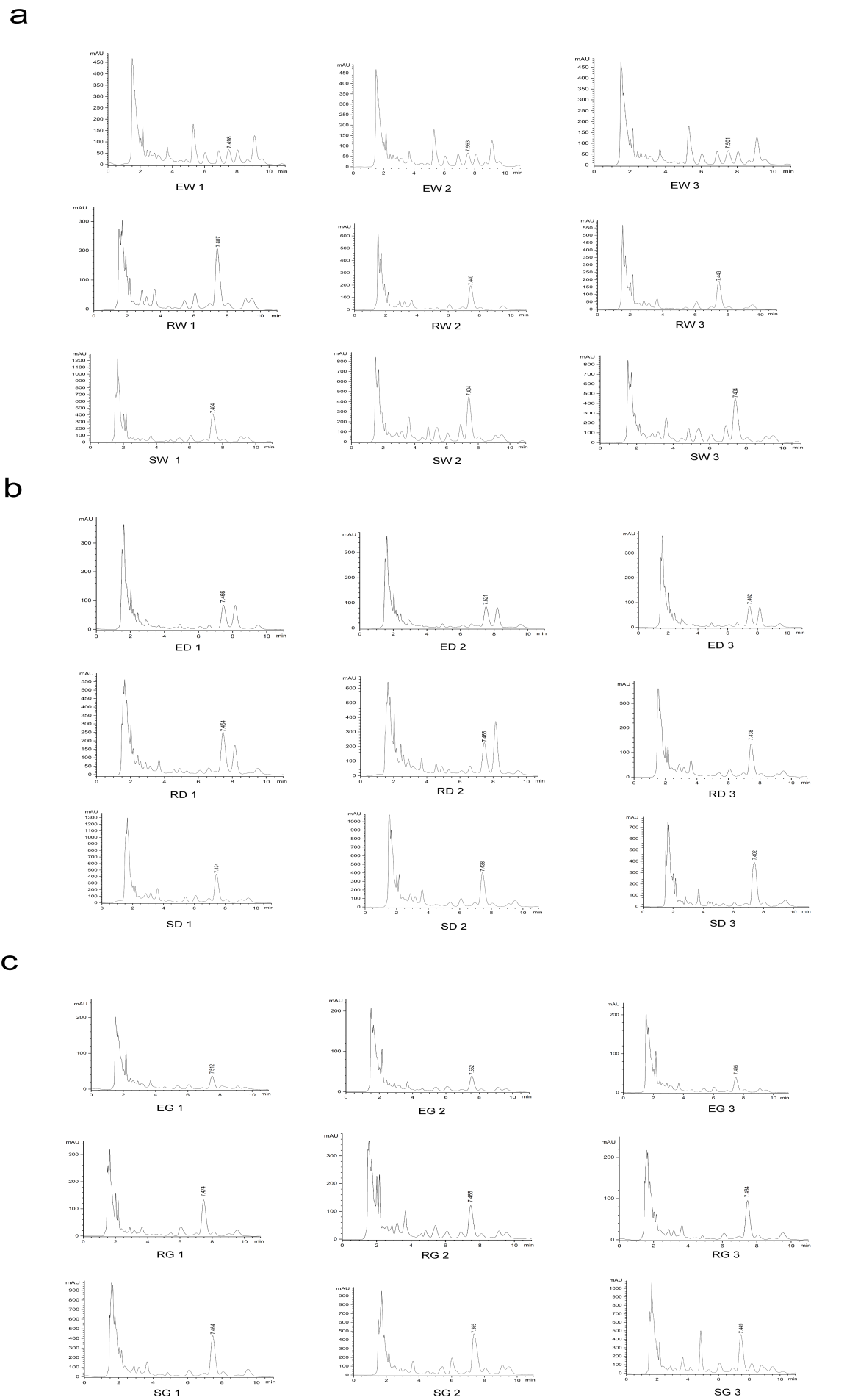


**Figure S2** glycyrrhizic acid content in the root of three licorices species

Description: glycyrrhizic acid content in the root of *Glycyrrhiza uralensis* (a), *Glycyrrhiza inflata* (b), and *Glycyrrhiza glabra* (c). Ordinate is peak height (mAU); Abscissa is retention time (min).

## Figure S3. liquiritin content in the root of three licorices species


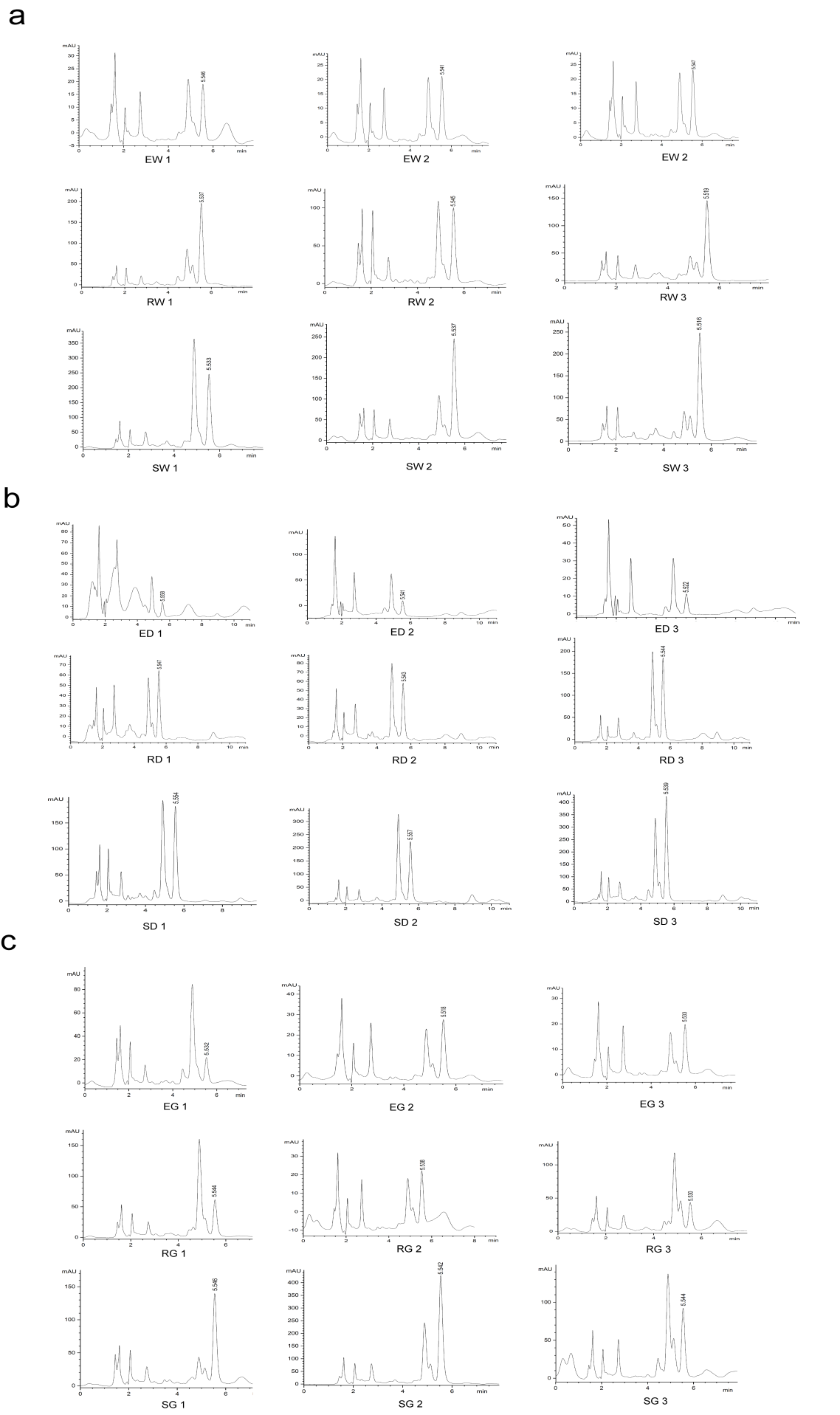


**Figure S3** liquiritin content in the root of three licorices species

Description: liquiritin content in the root of *Glycyrrhiza uralensis* (a), *Glycyrrhiza inflata* (b), and *Glycyrrhiza glabra* (c). Ordinate is peak height (mAU); Abscissa is retention time (min).

## Figure S4. PCR data of samples


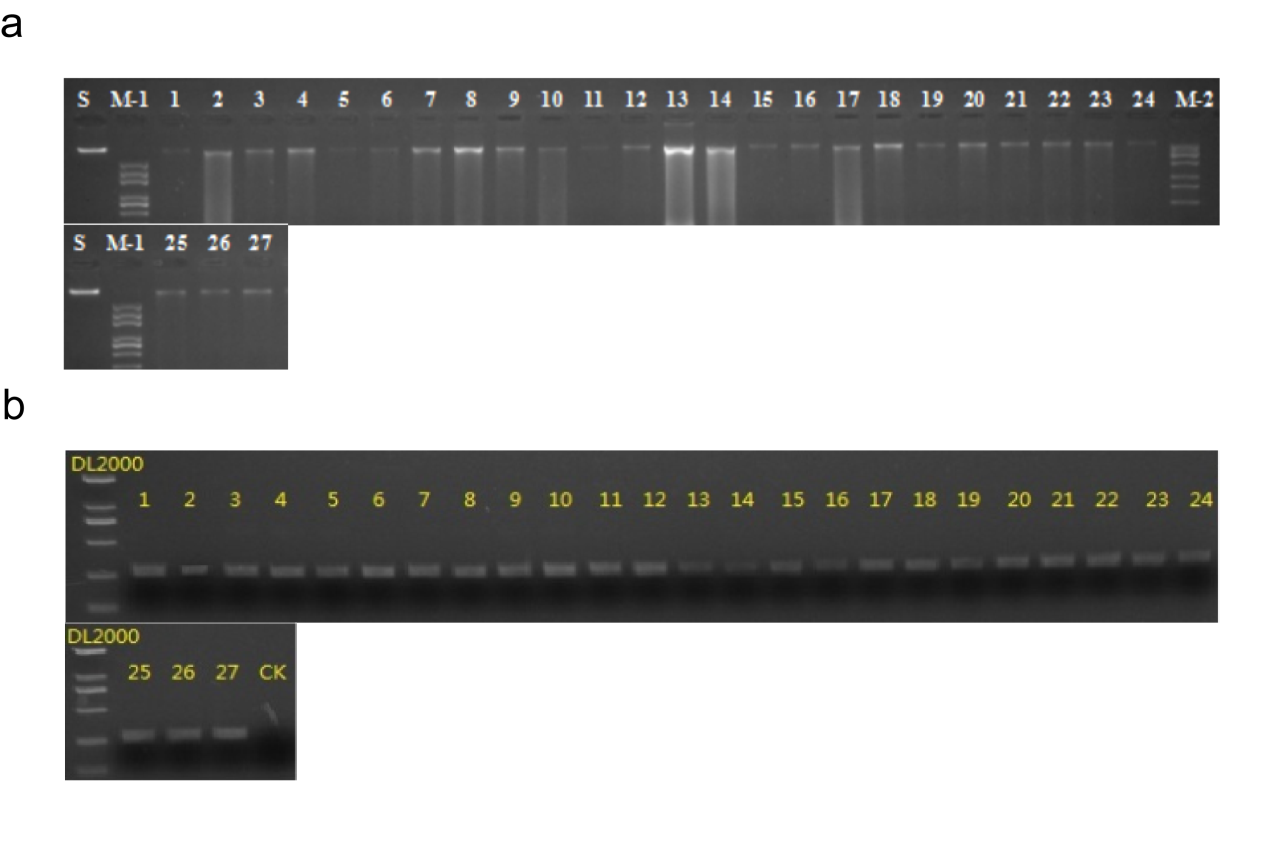


**Figure S4** PCR data of samples

Description: (a) is root samples; (b) is soil sample.
